# The CTLA-4 and PD-1/PD-L1 Inhibitory Pathways Independently Regulate Host Resistance to *Plasmodium*-induced Acute Immune Pathology

**DOI:** 10.1371/journal.ppat.1002504

**Published:** 2012-02-02

**Authors:** Julius Clemence R. Hafalla, Carla Claser, Kevin N. Couper, Georges Emile Grau, Laurent Renia, J. Brian de Souza, Eleanor M. Riley

**Affiliations:** 1 Department of Immunology and Infection, Faculty of Infectious and Tropical Diseases, London School of Hygiene and Tropical Medicine, London, United Kingdom; 2 Laboratory of Malaria Immunobiology, Singapore Immunology Network (SIgN), Agency for Science, Technology and Research (A*STAR), Biopolis, Singapore; 3 Vascular Immunology Unit, Department of Pathology, School of Medical Sciences, Bosch Institute, The University of Sydney, Camperdown, New South Wales, Australia; 4 Division of Infection and Immunity, University College London Medical School, London, United Kingdom; University of Pennsylvania, United States of America

## Abstract

The balance between pro-inflammatory and regulatory immune responses in determining optimal T cell activation is vital for the successful resolution of microbial infections. This balance is maintained in part by the negative regulators of T cell activation, CTLA-4 and PD-1/PD-L, which dampen effector responses during chronic infections. However, their role in acute infections, such as malaria, remains less clear. In this study, we determined the contribution of CTLA-4 and PD-1/PD-L to the regulation of T cell responses during *Plasmodium berghei* ANKA (*PbA*)-induced experimental cerebral malaria (ECM) in susceptible (C57BL/6) and resistant (BALB/c) mice. We found that the expression of CTLA-4 and PD-1 on T cells correlates with the extent of pro-inflammatory responses induced during *PbA* infection, being higher in C57BL/6 than in BALB/c mice. Thus, ECM develops despite high levels of expression of these inhibitory receptors. However, antibody-mediated blockade of either the CTLA-4 or PD-1/PD-L1, but not the PD-1/PD-L2, pathways during *PbA*-infection in ECM-resistant BALB/c mice resulted in higher levels of T cell activation, enhanced IFN-γ production, increased intravascular arrest of both parasitised erythrocytes and CD8^+^ T cells to the brain, and augmented incidence of ECM. Thus, in ECM-resistant BALB/c mice, CTLA-4 and PD-1/PD-L1 represent essential, independent and non-redundant pathways for maintaining T cell homeostasis during a virulent malaria infection. Moreover, neutralisation of IFN-γ or depletion of CD8^+^ T cells during *Pb*A infection was shown to reverse the pathologic effects of regulatory pathway blockade, highlighting that the aetiology of ECM in the BALB/c mice is similar to that in C57BL/6 mice. In summary, our results underscore the differential and complex regulation that governs immune responses to malaria parasites.

## Author Summary

T cells are part of the body's defense system in response to infection. However, once the infection has been suitably controlled, these T cells must be switched off. Inhibitory pathways, such as CTLA-4 and PD-1, are known to send the ‘turn off’ signal to T cells during chronic infections. However, their roles in acute infections, such as malaria, are unclear. We compared the function of these inhibitory pathways in mice that are either susceptible or resistant to severe malarial disease (cerebral malaria). Strikingly, we found that receptors for CTLA-4 and PD-1 are more highly expressed in T cells from susceptible mice than from resistant mice. Therefore, cerebral malaria develops despite the high expression of these inhibitory receptors. Moreover, we demonstrated that blocking these inhibitory receptors in the resistant mice increased the function of T cells, which in turn led to the characteristic signs of cerebral malaria. Finally, reminiscent of what is known for the susceptible strain, we confirmed that certain T cells (CD8^+^) and molecules (IFN-γ) are crucial to the development of cerebral malaria in the otherwise resistant mice. Thus, the CTLA-4 and PD-1 inhibitory pathways have essential, independent and non-redundant roles in regulating the body's complex response to malaria.

## Introduction

The outcome of microbial infections is dependent on the balance between pro-inflammatory and regulatory immune responses. Priming of naïve T cells and their differentiation into effector cells needs to be balanced by switching off these cells, at an appropriate stage of infection, in order to prevent tissue damage (immune pathology). Two important T cell inhibitory pathways involve signalling through members of the CD28:B7 superfamily of costimulatory molecules, namely cytotoxic T lymphocyte antigen-4 (CTLA-4; CD152) and programmed death-1 (PD-1; CD279). While CTLA-4 is expressed on activated T cells including regulatory T cells [1]–[3], PD-1 is broadly expressed on activated T cells, regulatory T cells and other haematopoietic cells [4]. T cell activation through the T cell receptor (TCR) and the costimulatory molecule CD28 results in increased expression of CTLA-4 [1]. Since both CD28 and CTLA-4 bind to B7-1 (CD80) and B7-2 (CD86) on antigen-presenting cells [5]–[7], sequential expression of CD28 and then CTLA-4 allows T cells to be intrinsically self-regulating. CTLA-4 has higher affinity to the B7 molecules than CD28. Similarly, PD-1 binds to PD ligand 1 (PD-L1; CD274) and 2 (PD-L1; CD273) which are upregulated on activated macrophages and dendritic cells (DCs) [4]. In addition, PD-L1, which is also expressed on activated T cells, has recently been shown to bind B7-1 [8], suggesting that there may be opportunities for cross-talk between the CTLA-4/B7 and PD-1/PD-L1 pathways. Consistent with their roles in the physiological regulation of cellular immune responses, *CTLA-4* ^−/−^ and *PD-1* ^−/−^ mice develop spontaneous autoimmune diseases; *CTLA-4* ^−/−^ mice die 2–3 weeks after birth from systemic lymphoproliferation [9], [10] while *PD-1* ^−/−^ mice develop lupus-like glomerulonephritis and destructive arthritis [11], [12].

Accumulating data suggest that PD-1/PD-L1 signalling, and in some cases CTLA-4 signalling, is implicated in the T cell exhaustion that is seen in many chronic infections [13]–[15]. For example, expression of PD-1 is associated with progressive loss of CD8^+^ T cell effector function during persistent lymphocytic choriomeningitis virus (LCMV) infection in mice; blockade of PD-1/PD-L1 interactions but not the CTLA-4 pathway augmented T cell function and allowed the virus to be controlled [16]. Similarly, in humans, PD-1 is upregulated on both CD4^+^ and CD8^+^ T cells during human immunodeficiency virus (HIV) infection and on CD8^+^ T cells during hepatitis C virus (HCV) infections and is associated with functional impairment of T cells and disease progression [17]–[19]. *In vitro* blockade of PD-1/PD-L1 pathway significantly increases CD8^+^ and CD4^+^ T cell function during HIV infection [20]. There is limited information available on the role of the PD-1/PD-L2 in chronic infections. *In vitro* blockade of PD-1/PD-L1 and to a lesser extent PD-1/PD-L2 resulted in reversal of immune dysfunction in HCV [21]. PD-L2 expression on dendritic cells is correlated to morbidity in experimental chronic schistosomiasis [22]. High levels of CTLA-4 expression are found on HIV-specific CD4^+^ T cells, but not on CD8+ T cells, and *in vitro* blockade of CTLA-4 enhances HIV-specific CD4^+^ T cell function [17]. Likewise, CTLA-4 blockade augments T cell responses to, and resolution of chronic infections such as *Helicobacter pylori* [23], *Leishmania donovani* [24], *Leishmania chagasi* [25] and *Trypanosoma cruzi* [26] in mice.

With regard to acute infections, CTLA-4 blockade during *Nippostrongylus brasiliensis* [27] and *Listeria monocytogenes* [28] infection greatly enhanced T cell responses, resulting in more effective infection control. However, although CTLA-4 blockade enhanced T cell responses during *Mycobacterium bovis* infection, this did not have any effect on bacterial clearance [29]. *PD-L1* ^−/−^ mice are markedly more resistant to rabies virus [30] and *Histoplasma capsulatum* infection [31] than are wild-type mice. While these studies clearly indicate a role for the PD-1/PD-L1 pathway in dampening T cell responses, there is, rather confusingly, some evidence that this pathway is important in promoting CD8^+^ T cell responses in murine influenza virus [32] and *Listeria monocytogenes* [33] infections, suggesting that the outcome of PD-1/PD-L1 interactions might be modified by other regulatory pathways. Moreover, in *Plasmodium yoelii* malaria infections, CTLA-4 blockade increased T cell activation and IFN-γ production leading to early resolution of infections with the non-lethal 17X strain, but to increased severity of infections with the highly virulent 17XL strain of the parasite [34], suggesting that enhancing T cell activation can be beneficial in relatively mild infections but can exacerbate virulent infections. Limited data are available for the PD-1/PD-L2 pathway during acute infections: PD-1/PD-L2 but not PD-1/PD-L1 blockade favours trypanosomatid growth in macrophages [35] and PD-L2 blockade enhances Th2 responses during *Nippostrongylus* infection [36].

Very few studies have directly contrasted the roles of CTLA-4 and PD-1 in the same infection, investigated the role of these pathways in determining susceptibility or resistance to infection in different mouse strains, or evaluated the extent to which they modulate immune pathology versus pathogen clearance. Here we have directly compared the roles of the CTLA-4 and PD-1 pathways in an acute malaria infection model in which resistance or susceptibility to immune-mediated pathology varies among strains of mice.

*P. berghei* ANKA (*PbA*) infection of experimental cerebral malaria (ECM)-susceptible C57BL/6 mice reproduces the neurological signs associated with human cerebral malaria, the most severe complication of infection by the human parasite, *P. falciparum* [37]. Ante-mortem, the diagnostic neurological signs of ECM are ataxia and/or paralysis, which quickly leads to seizures, prostration and death within 10 days of infection. Histologically, CM is characterised by oedema, petechial haemorrhages and adherence of leucocytes and parasitised red blood cells to brain endothelium [37]. The essential triggers for ECM include systemic priming of CD4^+^ T cells by conventional DCs [37], the production of pro-inflammatory cytokines such as IFN-γ [38], [39], the recruitment of effector CD8^+^ T cells to the brain [40], [41], and parasite accumulation in the cerebral microvasculature [42]–[44]. C57BL/6 mice infected with *Pb*A develop a multi-organ disease as recently described [45] and – as in the brain - this is mediated by T cells and IFN-γ. However, this systemic disease, in the absence of cerebral involvement, does not appear to be fatal and mice will die at a later time point from hyperparasitaemia. The current best model of the pathogenesis of ECM is that CD8^+^ T cells damage cerebral vascular endothelial cells and the underlying basement membrane, thereby breaching the blood brain barrier, causing haemorrhage and oedema. Thus, pathology manifests initially in the brain because this organ is particularly vulnerable to the immediate consequences of endothelial damage.

In contrast, the majority of *PbA*-infected BALB/c mice do not develop ECM but die from high parasitaemia and anaemia 2–3 weeks post-infection [46]. Hence, BALB/c mice are considered resistant to *PbA*-induced immune pathology. We hypothesised, therefore, that T cell-mediated inflammatory responses may be down-regulated in BALB/c mice (preventing control of parasitaemia but also preventing accumulation of T cells in the brain), and that this might be due to differential regulation of CD4^+^ and/or CD8^+^ T cells by CTLA-4 and/or PD-1. We found that in C57BL/6 mice, ECM develops despite high levels of expression of inhibitory receptors on CD4^+^ and CD8^+^ T cells. Conversely, we found that blockade of either CTLA-4 or PD-1/PD-L1, but not PD-1/PD-L2, during *PbA*-infection leads to the onset of ECM in normally resistant BALB/c mice and that this is accompanied by the characteristic features of T cell hyperactivity, raised IFN-γ levels and accumulation of CD8^+^ T cells and parasites in the brain. Thus, the CTLA-4 and PD-1/PD-L1 pathways seem to function very efficiently in BALB/c mice, maintaining the balance between immunity and immune pathology during the critical early stage of infection.

## Materials and Methods

### Ethics statement

Animal experiments performed in the United Kingdom were approved by the LSHTM Animal Procedures and Ethics Committee and were performed under licence from the United Kingdom Home Office under the Animals (Scientific Procedures) Act 1986. In Singapore, all experiments and procedures were approved by the Institutional Animal Care and Use Committee (IACUC) of A*STAR (Biopolis, Singapore) (Authorization No IACUC 080321) in accordance with the guidelines of the Agri-Food and Veterinary Authority (AVA) and the National Advisory Committee for Laboratory Animal Research (NACLAR) of Singapore.

### Mice, parasites and experimental infections

Six- to twelve week old BALB/cAnNCrl mice and C57BL/6NCrl mice were purchased from Charles River UK Ltd and maintained under barrier conditions. *Pb*A parasites, derived from the *Pb*A clone 15cy1, which had been genetically engineered to express green fluorescent protein (*Pb*A*gfp* [47], referred to here as *Pb*A) were maintained by passage through naïve mice. For *in vivo* imaging experiments, seven- to eight weeks old BALB/cJ mice were bred in-house and kept under specific pathogen-free conditions. Transgenic *P. berghei* ANKA 231c1l parasites expressing luciferase under the control of the ef1-a promoter (referred here as *Pb*Aluc) were provided by Dr. Christian Engwerda (Queensland Institute for Medical Research, Brisbane, Australia) from a stock originally from Leiden, The Netherlands [48], [49].

Experimental infections were initiated by i.v. inoculation of 10^4^ *Pb*A-parasitised red blood cells (pRBC) and infected mice were monitored for neurological symptoms (paralysis, ataxia, convulsions, and coma occurring between day 6 and 10 post-infection) as previously described [50]. All mice that developed signs of irreversible pathology were immediately humanely sacrificed and their brains examined for signs of ECM (see Protocol S1 in Text S1 for additional details). Cumulative ECM incidence during the observation period was then reported. Parasitaemia was determined by examination of Giemsa-stained thin blood smears. On various days post-infection, mice were sacrificed and exsanguinated. Moreover, their spleens were removed, and single spleen cell suspensions were prepared by homogenisation through a 70 µm cell strainer (BD Biosciences). CD4^+^ and CD8^+^ T cells were purified by magnetic bead sorting (MACS, Miltenyi Biotec). Brain-sequestered leucocytes were isolated from perfused animals as described [51]. Live cells were counted by trypan blue exclusion. Heparinised plasma was stored at −70°C for cytokine quantification.

### *In vivo* administration of antibodies

Blocking antibodies to CTLA-4 [UC10-4F10-11], PD-L1 [9G2] and PD-L2 [TY5] and neutralising antibodies to IFN-γ [XMG1.2] and TNF [XT3.11] were administered by intraperitoneal injection (0.4 mg/mouse) on days −1, 1, 3, 5 and 7 of infection. Depleting antibodies to CD4 [GK1.5] and CD8 [53.6.72] were administered by intraperitoneal injection (0.25 mg/mouse) on days −1, 1, 4 and 6 (or on days 4 and 6) of infection. All antibodies were rat-α-mouse IgG and were obtained from BioXCell; control rat IgG was obtained from Pierce.

### Flow cytometry

Antibodies [clones] for cell-surface staining were obtained from eBiosciences (α-mouse CD4 [GK1.5], CD8 [53.6-7], CD11a [M17/4], CD11c [N418], CD44 [IM7], CD62L [MEL-14], CD71 [R17217], CD273/PD-L2 [122], CD274/PD-L1 [MIH5], CD279/PD-1 [RMP1-30]), F4/80 [BM8] or BD Biosciences (α-mouse CD3 [145-2C11], CD4 [RM4-5] and CD8 [53-6.7]). Isolated leucocytes were directly stained according to standard protocols. Antibodies for intracellular staining were obtained from eBiosciences (α-mouse CD152/CTLA4 [UC10-4B9], FoxP3 [FJK-16s], and IFN-γ [XMG1.2]). Intracellular staining was performed by permeabilising cells with 0.1% Saponin/PBS. Cells were analysed using a FACSCalibur or LSR II (BD Immunocytometry Systems) and FlowJo software (TreeStar).

### Cytokine quantification

Plasma cytokines were assayed by cytometric bead array (mouse inflammation kit; BD Bioscience) following the manufacturer's protocol. Intracellular IFN-γ was assayed by flow cytometry (above) following 5-hour culture of mixed spleen cells in the presence of PMA (50 ng/mL), ionomycin (1 µg/mL), and Brefeldin A (1 µg/mL). Secreted IFN-γ and IL-10 were assayed by conventional ELISA [52] in supernatants of purified CD4^+^ or CD8^+^ T cells cultured (at 10^5^ cells per well) for 24 or 48 hours respectively in the presence of α-CD3 [clone 145-2C11, 1 µg/mL] and α-CD28 [clone 37.51, 1 µg/mL] antibodies (eBioscience).

### Histopathology

Brain and liver tissues were fixed in 10% formaldehyde saline, paraffin-wax embedded, sectioned, stained with haematoxylin and eosin and examined by light microscopy at 20X magnification.

### Bioluminescent imaging

Distribution of *Pb*Aluc parasites was monitored daily by *in vivo* imaging (IVIS; Xenogen, Alameda, California). Infected mice were anaesthesised, injected s.c. with 100 µl of D-luciferin potassium salt (Caliper Life Sciences) (5mg/ml in PBS) and, two minutes later, bioluminescence images were acquired, with medium binning factor and fields-of-view (FOV) of 21.7 and 4 cm for the whole body (ventral) and head (dorsal), respectively. Imaging time was between 5 to 60 seconds per mouse. In terminal experiments, mice were given a second injection of luciferase substrate and, within 3 minutes, mice were sacrificed (by cervical dislocation). Brains were removed and imaged with 10 cm FOV. To allow comparison of images from different days of the experiment, uninfected mice injected with luciferin were imaged for background subtraction. Bioluminescence in the brains was quantified using Living Imaging 3.0 software and expressed as average radiance units (p/s/cm^2^/sr).

### Statistical analysis

Statistical analysis was performed in GraphPad Prism (GraphPad Software Inc). Comparisons between two groups were made using the Mann Whitney test. For comparisons involving more than two groups, statistical significance was determined using the Kruksal Wallis test with Dunn's post-test for multiple comparisons with p<0.05 taken as evidence of a significant difference. Differences in survival curves between two groups were analysed using the Log-rank (Mantel Cox) test. Differences in cumulative ECM incidence between two groups was analysed using the Fisher's exact test. Bonferroni correction was used to adjust for multiple comparisons within the Log-rank (Mantel Cox) and Fisher's exact tests. The Bonferroni-corrected threshold for significance was calculated by dividing the conventionally set level of significance (0.05) by the number of comparisons.

## Results

### Expression of the inhibitory receptors CTLA-4 and PD-1 correlates with the induction of pro-inflammatory responses during PbA infection in both ECM-resistant and ECM-susceptible mouse strains

**Figure 1:**
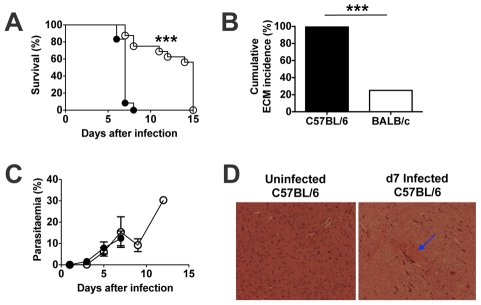
Course of infection of *Plasmodium berghei* ANKA (PbA) in C5BL/6 (ECM-susceptible) and BALB/c (ECM-resistant) mice. Mice were infected i.v. with 10^4^ *PbA* pRBCs. The course of infection in C57BL/6 (n = 12) and BALB/c (n = 16) mice was followed by monitoring: (**A**) Cumulative survival – C57BL/6 (•) and BALB/c (○), *** P<0.0001 (Log-rank (Mantel Cox) test); and (**B**) Development of experimental cerebral malaria (ECM; cumulative incidence during the observation period), *** P<0.0001 (Fisher's exact test). The incidence of ECM was based on neurological signs, i.e. ataxia and paralysis. This was confirmed by histopathological examination of the brain. Surviving BALB/c mice were euthanized on day 15 due to the development of high parasitaemia and anemia; these mice were not ataxic or paralysed and they did not have brain lesions. (**C**) Parasitaemias are shown as mean + SD; representative of three experiments (four to six mice per group in each experiment). (**D**) H&E histopathology of brains from uninfected (left panel) and day 7 infected (right panel) C57BL/6 mice. Blue arrow indicates an area of haemorrhage. Magnification = 20X.

The course of *Pb*A infection was compared in C57BL/6 and BALB/c mice (**Figure 1A**). *PbA*-infected C57BL/6 mice became moribund on day 7 post-infection after developing neurological signs. In contrast, the majority of *PbA*-infected BALB/c mice survived up to day 15 post-infection when they were euthanised due to the development of severe anaemia and moderate parasitaemia. Over the course of three experiments, 100% (12/12) of C57BL/6 mice but only 25% (4/16) of BALB/c mice developed signs of ECM (**Figure 1B**). There was no difference between C57BL/6 and BALB/c mice in peripheral parasitaemia up to day 7 (**Figure 1C**).

**Figure 2:**
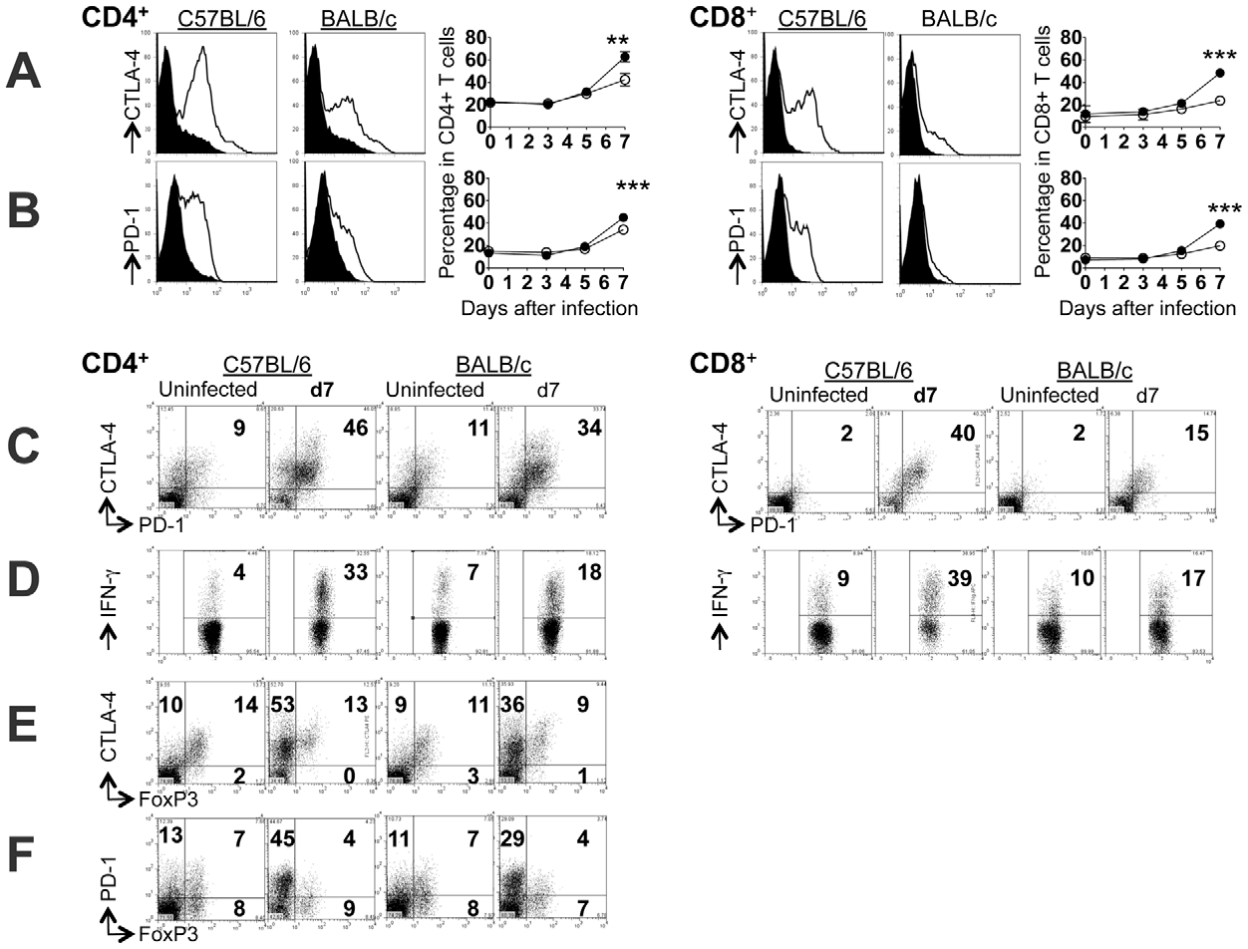
Differential expression of CTLA-4 and PD-1 by T cells of *Pb*A-infected C5BL/6 and BALB/c mice. Mice were infected i.v. with 10^4^ *PbA* pRBCs. Splenocytes were prepared from uninfected (solid curves) or day 7 infected (clear curves) mice and stained for CD4 (left) or CD8 (right) expression of (**A**) CTLA-4 or (**B**) PD-1. For CTLA-4, staining was for both surface and intracellular protein; for PD-1, staining was for surface expression only. Line graphs show the kinetics of expression during the first 7 days of infection: data points (mean ± SD) from C57BL/6 (•) and BALB/c (○); n = 6, ** P<0.01 and *** P<0.001, P-values (Mann Whitney U test). (**C**) CD4^+^ and CD8^+^ T cells from uninfected and day 7 infected mice were stained for intracellular IFN-γ following stimulation with PMA/Ionomycin in the presence of Brefeldin A. CD4^+^ T cells were directly stained *ex vivo* for (**D**) intracellular CTLA-4 and surface PD-1; (**E**) CTLA-4 and intracellular FoxP3 or (**F**) surface PD-1 and intracellular FoxP3. Data for (C) and (D) are representative of three independent experiments with three to six mice in each group. Data for (E) and (F) are representative of two independent experiments with three mice in each group.

To determine if T cell regulatory receptors could explain differences in susceptibility to ECM, intracellular expression of CTLA-4 and surface expression of PD-1 were compared on splenic T cells of BALB/c and C57BL/6 mice at different times after *Pb*A infection (**Figure 2**). The proportions of CD4^+^ and CD8^+^ T cells expressing either CTLA-4 or PD-1 were similar in the two mouse strains during the first 6 days of infection, but surprisingly, on day 7 – i.e. at exactly the time when susceptible mice began to show signs of ECM – the proportions of both CD4^+^ and CD8^+^ T cells expressing CTLA-4 and PD-1 were significantly higher in ECM-susceptible C75BL/6 mice than in ECM-resistant BALB/c mice (**Figure 2A, B**). Consistent with previous reports in other models that PD-L1 is also expressed on T cells and is further upregulated during activation [4], PD-L1 was upregulated on virtually all CD4^+^ and CD8^+^ T cells by day 5 of *Pb*A infection in both C57BL/6 and BALB/c mice (Figure S1A in Text S1). In addition, dendritic cells (Figure S1B in Text S1) and macrophages (Figure S1C in Text S1) from both groups of mice upregulated PD-L1 and PD-L2 during infection.

The higher frequency of CTLA-4^hi^ and PD-1^hi^ cells in infected C57BL/6 mice correlated with a higher proportion of CD4^+^ and CD8^+^ splenic T cells able to secrete IFN-γ (**Figure 2C**). Furthermore, T cells from C57BL/6 mice expressed significantly higher levels of effector and activation markers: CD11a^hi^, a surrogate marker for polyclonal, antigen-experienced CD8^+^ T cells [53], [54] and CD62L^lo^, a commonly used marker of effector cells [53], [55] (Figure S2 in Text S1).

In both strains of infected mice, and in both CD4^+^ and CD8^+^ T cells, CTLA-4 and PD-1 seem to be co-expressed on the same cells (**Figure 2D**, Figure S3 in Text S1), indicating that the two receptors may function co-operatively. Consequently, CTLA-4^hi^/PD-1^hi^ expression on T cells coincided with CD11a^hi^ (Figure S3B,E in Text S1) and CD62L^lo^ (Figure S3C,F in Text S1) suggesting that these are activated cells induced in response to infection. Moreover, the vast majority of CTLA-4^hi^ and PD-1^hi^ CD4^+^ T cells were FoxP3^-^, indicating that they are not classical regulatory T cells (**Figure 2E,F**).

Together, these data indicate that CTLA-4, PD-1 and PD-L1 are upregulated on activated T cells during *PbA* infection in both ECM-susceptible and ECM-resistant mice. However, the very high degree of T cell activation in C57BL/6 mice may provide a situation where positive signals override physiological levels of immune regulation mediated by CTLA-4 and PD-1 such that they are unable to prevent immune-mediated pathology.

### Blockade of CTLA-4 and PD-1/PD-L1 pathways induces experimental cerebral malaria in otherwise resistant mice

To determine whether the CTLA-4 and PD-1 pathways modulate ECM pathogenesis in the ECM-resistant strain, the outcome of *Pb*A infection was compared between control mice and mice treated with blocking antibodies to PD-L1 or CTLA-4. We decided to focus on CTLA-4 and PD-1/PD-L1 particularly because both of these inhibitory pathways have been extensively studied in chronic infections but a comparison of the two pathways during an acute infection was lacking.

**Figure 3:**
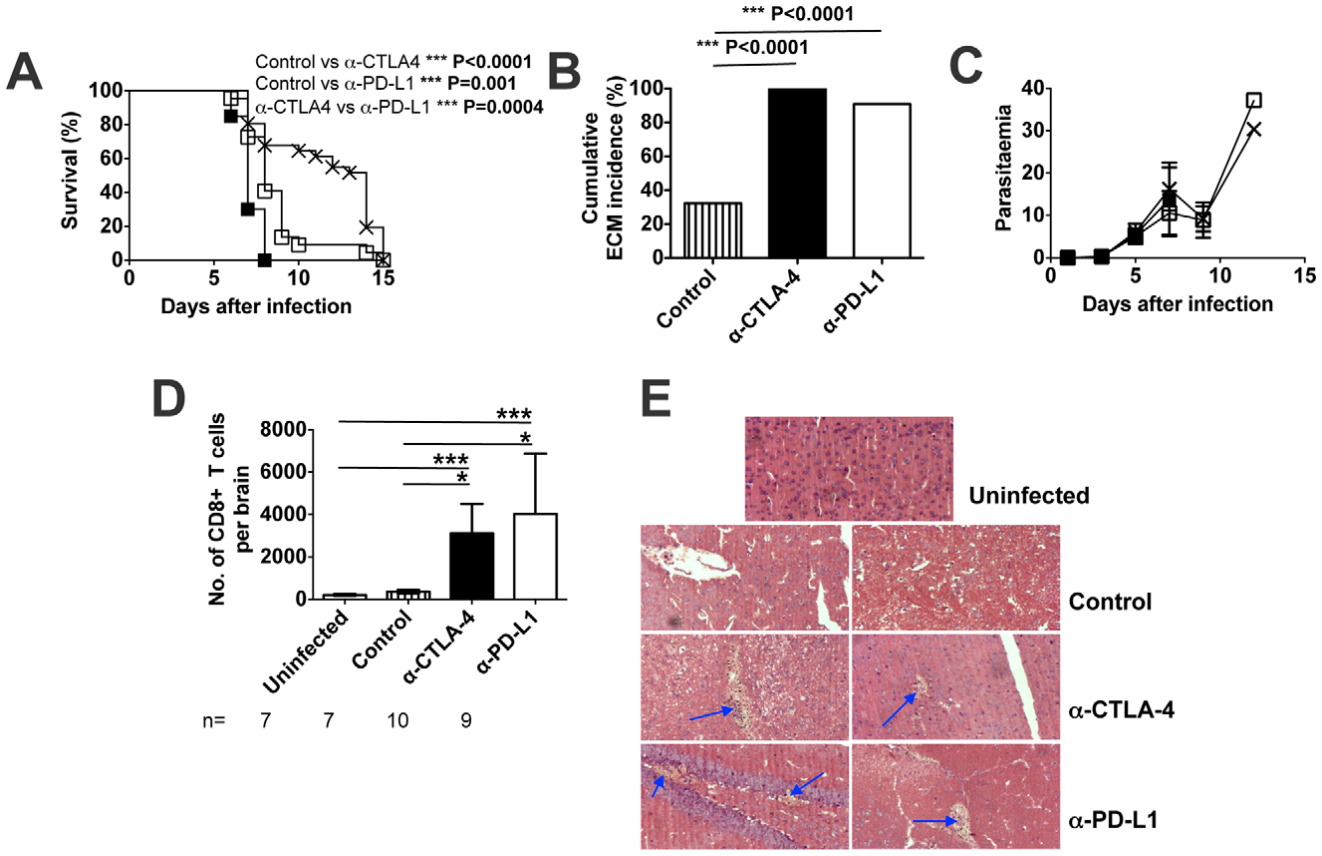
CTLA-4 and PD-1 blockade in *Pb*A-infected BALB/c mice leads to onset of ECM. BALB/c mice were infected i.v. with 10^4^ *PbA* pRBCs and treated with α-CTLA4 or α-PD-L1 antibodies or with no antibody. (**A**) Cumulative survival curve: X = Control (n = 31); ▪ = α-CTLA-4 (n = 20); □ = α-PD-L1 (n = 22), P-values (Log-rank (Mantel Cox) test). (**B**) Cumulative incidence of mice developing ECM, P-values (Fisher's exact) test. Bonferroni correction was used to adjust for multiple comparisons; threshold for significance is P<0.017 for (A) and (B). The incidence of ECM was based on neurological signs, i.e. ataxia and paralysis. Surviving BALB/c mice were euthanized on day 15 due to high parasitaemia and anemia. (**C**) Parasitaemia levels, shown as mean ± SD, of *PbA*-infected mice: X = Control; ▪ = α-CTLA-4; □ = α-PD-L1. Data are representative of three independent experiments with four to six mice in each group. (**D**) Absolute numbers of brain infiltrating CD8 T cell lymphocytes. Data (mean ± SD) are from two experiments, the numbers of animals are shown; P-values (Kruskal-Wallis Test/Dunn's multiple comparison test). (**E**) Histological examination of H&E stained brain sections from uninfected and day 7 infected mice (control without ECM and treated with ECM). Blue arrows indicate areas of haemorrhages. Magnification = 20X.

BALB/c mice treated with either α-CTLA-4 (20/20; 100%) or α-PD-L1 (20/22; 90.9%) developed classical neurological signs of ECM and were euthanised on days 7–8 post-infection or days 8–10 post-infection, respectively, whereas control mice (treated with rat IgG or PBS) survived for up to two weeks and were euthanised due to severe anaemia **(** **Figure 3A, B**). Importantly, antibody treatment had no effect on parasitaemia (**Figure 3C**). The survival curves for α-CTLA-4 and α-PD-L1-treated mice differ significantly from the control mice. In addition, the survival curves for α-CTLA-4 and α-PD-L1-treated mice were also significantly different from each other. Since PD-1 also binds another ligand, PD-L2, the outcome of *Pb*A infection was compared between control mice and mice treated with α-PD-L2. As shown in Figure S4 in Text S1, α-PD-L2 treatment had no effect on the course of infection or the pathological outcome of *Pb*A-infection in BALB/c mice.

Consistent with their development of neurological signs of ECM, numbers of arrested CD8^+^ T cells were significantly higher in brain microvessels of α-CTLA-4-treated and α-PD-L1-treated BALB/c mice than in brains of control mice (**Figure 3D**). Moreover, histological examination revealed more frequent petechial haemorrhages and a higher proportion of cerebral blood vessels plugged with parasitised red blood cells in brains of BALB/c mice treated with α-CTLA-4 or α-PD-L1 antibodies than in brains of control (treated with rat IgG or PBS or left untreated) mice (**Figure 3E** **,** **Table 1**) as well as increased numbers of pigmented (parasite-containing) macrophages in their livers (**Table 1**).

**Table 1:** Histological analysis of brain and liver sections.

	Brain		Liver
	Petechial haemorrhages/50 fields^1^	Plugged vessels/50 fields^2^	Pigmented macrophages/50 fields^3^
Control (untreated)	3.8±5.9	+	153±6
α-CTLA-4	46.0±10.2	++/+++	263±33
α-PD-L1	24.9±4.8	+	218±11

1 Sections from each mouse were examined and the numbers in 50 fields were recorded.

Control vs α-CTLA-4 p<0.05, Control vs α-PD-L1 p<0.05, α-CTLA-4 vs α-PD-L1 p<0.05.

2 Vessels plugged with pRBCs and leucocytes/50 fields: +++ = 11–15, ++ = 6–10, + = 1–5.

3 Control vs α-CTLA-4 p = 0.01, Control vs α-PD-L1 p = 0.02, α-CTLA-4 vs α-PD-L1 p = 0.07.

**Figure 4:**
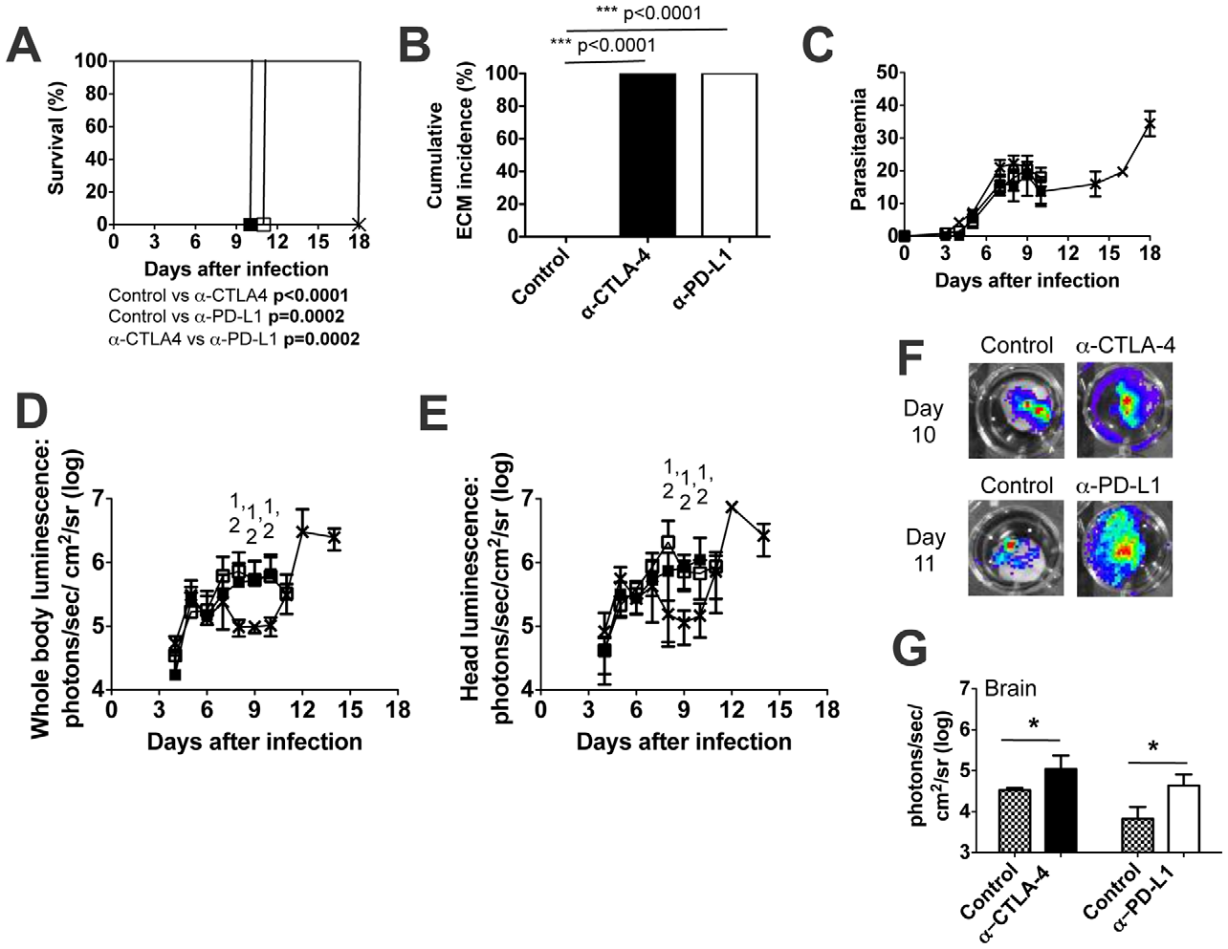
Onset of ECM after CTLA-4 and PD-1 blockade in *Pb*A-infected BALB/c mice is associated with parasite accumulation in the brain. BALB/c mice were infected i.v. with 10^4^ *PbA*luc pRBCs and were either untreated (control) or treated with α-CTLA4 or α-PD-L1 antibodies. (A) Cumulative survival curve: X = Control (n = 10); ▪ = α-CTLA-4 (n = 10); □ = α-PD-L1 (n = 10), P-values (Log-rank (Mantel Cox) test). (B) Cumulative incidence of mice developing ECM, P-values (Fisher's exact) test. Bonferroni correction was used to adjust for multiple comparisons; threshold for significance is P<0.017 for (A) and (B). (**C**) Parasitaemia, shown as mean ± SD, of *PbA*-infected mice: X = Control; ▪ = α-CTLA-4; □ = α-PD-L1. Data are representative of two independent experiments performed with 5 mice in each group. (**D–G**) Kinetics of parasite accumulation in the (**D**) whole body, (**E**) head and (**F,G**) isolated brain as measured by luciferase activity. X = Control; ▪ = α-CTLA-4; □ = α-PD-L1. For D-G, data are shown as mean ± SD. For **D** and **E**, ^1^ Control vs α-CTLA4 p<0.05, ^2^ Control vs α-PD-L1 p<0.05, and ^3^ α-CTLA4 vs a-PD-L1 p<0.05 (Kruskal-Wallis Test/Dunn's multiple comparison test). For **G**, P-values (Mann Whitney U test).

The accumulation of parasitised erythrocytes in the microvasculature of the brain is a cardinal feature of cerebral pathology in both human cerebral malaria [56] and ECM in mice [42], [43]. To further quantify the effects of CTLA-4 and PD-L1 blockade on parasite accumulation, α-CTLA-4- and α-PD-L1 antibody-treated BALB/c mice (and controls) were infected with *Pb*Aluc (transgenic *Pb*A expressing luciferase) and whole body, head and brain parasite burdens were quantified by bioluminescence at the onset of signs of ECM. The course of *Pb*Aluc infection in control, α-PD-L1- and α-CTLA-4-treated mice (**Figure 4A–C**) was similar to the course of *Pb*A infection (Figure 3A–C), confirming that insertion of the luciferase gene had not significantly altered the basic biology of the parasite, although the onset of ECM was slightly delayed (α-CTLA-4-treated mice developed ECM on day 10 post infection; α-PD-L1-teated mice developed ECM on day 11 and control mice were euthanised on day 18). Nevertheless, after day 7 of infection, whole body (**Figure 4D**), head (**Figure 4E**) and isolated brain (**Figure 4F,G**) parasite burdens were significantly higher in α-CTLA-4- and α-PD-L1-treated mice than in control mice. It should be noted that the detection of a weak luminescence signal in the brains of control (no ECM) mice in Figure 4F simply reflects the presence of luminescent parasites in the circulating blood in all organs. These results show that, despite similar peripheral parasite burden in control and antibody-treated mice, overall parasite biomass is significantly increased when the CTLA-4/PD-1 regulatory pathways are blocked.

Taken together, these data indicate that blockade of either the inhibitory receptor CTLA-4 or PD-L1, leads to striking immune pathology, with all the phenotypic characteristics of ECM, in otherwise ECM-resistant mice.

### Effector responses are enhanced following CTLA-4 and PD-L1 blockade

Since ECM is known to be a consequence of T cell-mediated inflammation in susceptible C57BL/6 mice[57], the development of neurological signs of ECM - together with CD8^+^ T cell infiltration and parasite accumulation in the microvasculature of the brain - in *PbA*-infected BALB/c mice after α-CTLA-4 or α-PD-L1 antibody treatment was suggestive of an enhanced inflammatory T cell response. To explore this hypothesis, we assessed levels of splenic T cell activation on day 7 post-infection (Figure S5 in Text S1). The proportions of splenic CD4^+^ and CD8^+^ T cells expressing an activated phenotype [CD71^+^ (transferrin receptor) and CD4^+^ T cells expressing CD44^+^ (Pgp-1)] were generally higher in α-CTLA-4-treated than in control mice (Figure S5 in Text S1). Notably, the proportions of splenic CD8^+^ T cells expressing CD11a^+^ was generally higher in both α-CTLA-4 and α-PD-L1-treated mice than in control mice. A trend was observed for higher proportions of splenic CD8^+^ T cells to express CD62L^−^ and CD11a^+^ in α-PD-L1-treated than in control mice. Thus, the slightly earlier onset of ECM in the α-CTLA-4-treated mice than in the α-PD-L1-treated mice (Figures 3A and 4A) correlates with the slightly higher levels (statistically significant) of T cell activation in the α-CTLA-4-treated mice.

### Indicators of systemic inflammation following CTLA-4 and PD-L1 blockade

**Figure 5:**
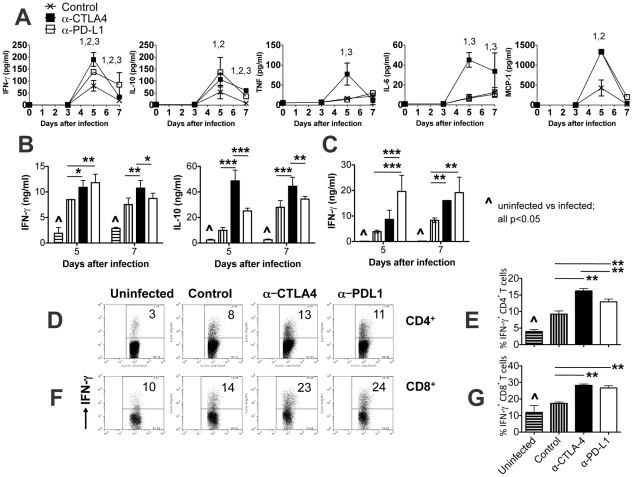
Enhanced cytokine secretion in *PbA*-infected BALB/c mice treated with α-CTLA and α-PD-L1 antibodies. (**A**) Plasma cytokine levels as determined by cytometric bead array: X = Control; ▪ = α-CTLA-4-treated; □ = α-PD-L1-treated. ^1^ Control vs α-CTLA4 p<0.05, ^2^ Control vs α-PD-L1 p<0.05, and ^3^ α-CTLA4 vs a-PD-L1 p<0.05. (**B,C**) IFN-γ and IL-10 ELISA of culture supernatants of splenocytes (from day 5 and day 7 infected mice) cultured for 48 h with α-CD3/CD28 antibodies. Spleens from 3–5 individual mice in each treatment group were pooled and each supernatant was analysed in triplicate. Mean (SE) values are shown for six wells. Uninfected = bar with horizontal line, Control = bar with vertical line, α-CTLA-4 = black bar, α-PD-L1 = white bar. (**D–G**) Intracellular IFN-γ staining of splenocytes isolated on day 7 post-infection and stimulated with PMA/Ionomycin for 5 hours in the presence of Brefeldin A. (**D,F**) Representative plots. (**E,G**) IFN-γ levels, shown as mean ± SD, are representative of three experiments (three to six mice per group in each experiment).

Plasma concentrations of cytokines and chemokines (measures of systemic inflammation) peaked on day 5 after infection (**Figure 5**). Plasma concentrations of IFN-γ, MCP-1 and IL-10 were significantly higher in α-CTLA-4- and α-PD-L1-treated mice than in untreated control mice. Concentrations of TNF and IL-6 were significantly higher in α-CTLA-4-treated mice than in either of the other two groups, and importantly, TNF and IL-6 levels did not differ between control mice and α-PD-L1-treated mice (**Figure 5A**).

To determine whether differences in plasma cytokine levels were related to differences in CD4^+^ and CD8^+^ T cell cytokine secretion, IFN-γ and IL-10 were quantified in culture supernatants of purified CD4^+^ and CD8^+^ T cells stimulated for 48 and 24 hours, respectively with α-CD3/α-CD28 antibodies. CD4^+^ T cells isolated on days 5 and 7 post-infection from α-CTLA-4-treated mice secreted significantly more IFN-γ and IL-10 than did CD4^+^ T cells from control mice (**Figure 5B**). In addition, CD4+ T cells isolated from day 5 post-infection from α-PD-L1-treated mice secreted more IFN-γ than did CD4+ T cells from control mice. Furthermore, CD4^+^ T cells isolated from α-CTLA-4-treated mice secreted significantly more IFN-γ (day 7) and IL-10 (days 5 and 7) than did CD4^+^ T cells from α-PD-L1 treated mice. Similarly, CD8^+^ T cells isolated from α-CTLA-4 (day 7 post-infection)- and α-PD-L1 (days 5 and 7 post-infection)-treated mice secreted significantly more IFN-γ than did CD8^+^ T cells from control mice (**Figure 5C**). CD8^+^ T cells isolated on day 5 post infection from α-PD-L1 -treated mice secreted significantly more IFN-γ than did CD8^+^ T cells from α-CTLA-4 treated mice. As further confirmation that the changes in IFN-γsecretion were due to changes in T cell function, spleen cells collected from treated mice on day 7 of infection were analysed by intracellular cytokine staining following short-term stimulation with PMA/ionomycin (**Figure 5D–G**). Consistent with the secreted cytokine data (above) the proportions of IFN-γ^+^splenic CD4^+^ and CD8^+^ T cells were higher in α-CTLA-4- and α-PD-L1-treated mice than in control mice.

### Development of ECM in BALB/c mice following α-CTLA-4 and α-PD-L1 blockade is T cell-mediated

**Figure 6:**
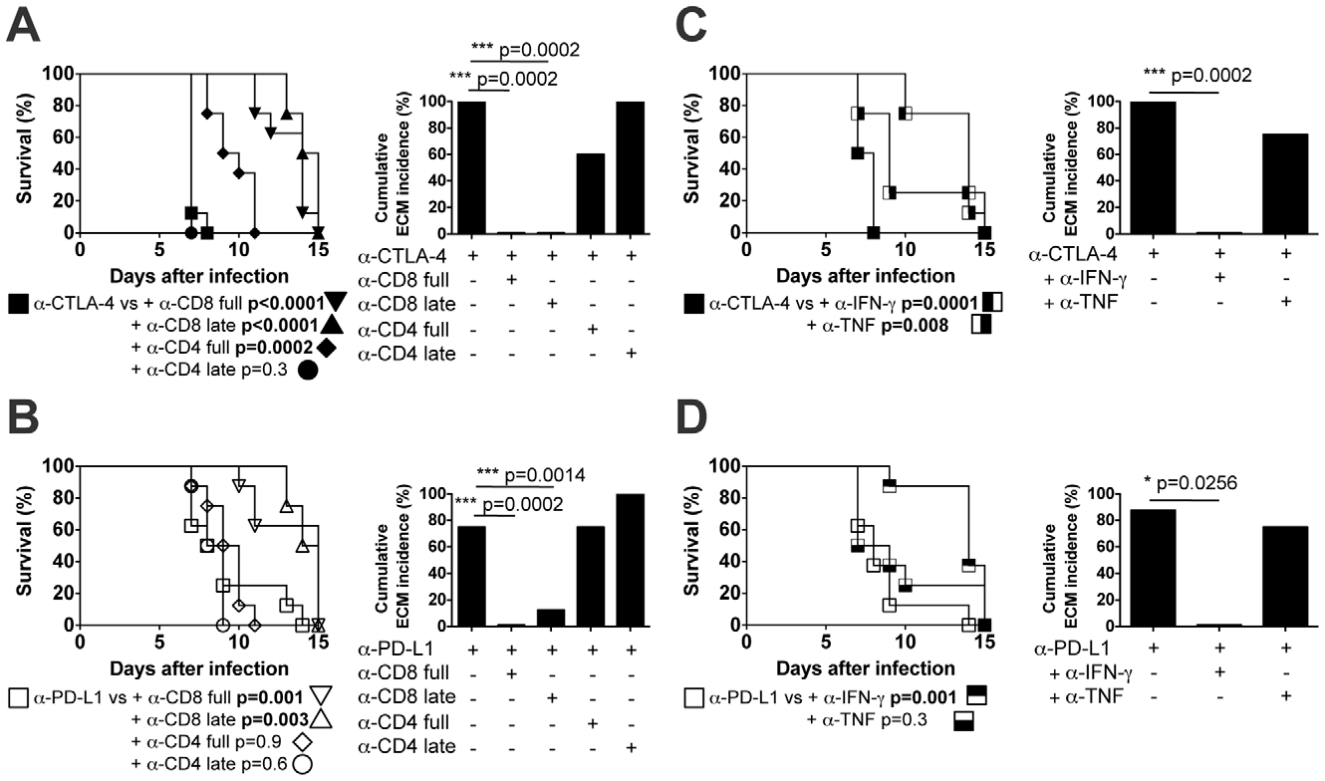
T cell depletion or cytokine blockade abrogates the effects of CTLA-4 and PD-L1 blockade in *PbA*-infected BALB/c mice. BALB/c mice were infected i.v. with 10^4^ *PbA* pRBCs and were treated with (**A,C**) α-CTLA or (**B,D**) α-PD-L1 antibodies. (**A,B**) CTLA-4 or PD-L1 blockade without T cell depletion (□,▪); with α-CD8 depletion throughout infection (▽,▾); with α-CD8 depletion early in infection (△,▴); with α-CD4 depletion throughout infection (◊, ⧫); or with α-CD4 depletion early in infection (○,•). Cumulative survival curves (left) and cumulative incidence of ECM (right). (**C,D**) CTLA-4 or PD-L1 blockade without cytokine blockade (□,▪) or with α-IFN-γ (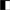) or α-TNF (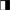) treatment on on days −1, 1, 3, 5 and 7. Cumulative survival curves (left) and cumulative incidence of ECM (right). Data are representative of two independent experiments performed with four mice in each group. P values: Log-rank (Mantel Cox) test for the survival curves, and Fisher's exact test for cumulative ECM incidence. Bonferroni correction was used to adjust for multiple comparisons; threshold for significance is P<0.01 for the T cell depletions and P<0.017 for the cytokine neutralisations.

Blockade of either CTLA-4 or PD-L1 renders normally resistant BALB/c mice fully susceptible to ECM, and this is associated with increased levels of activation and inflammatory cytokine secretion in both the CD4^+^ and CD8^+^ T cell populations, consistent with the hypothesis that signalling through both the CTLA-4 and PD-1 pathways is required to down-regulate T cell reactivity and thereby prevent ECM. To determine whether CD4^+^ or CD8^+^ T cell populations (or both) are the targets of CTLA-4 and PD-1 mediated regulation, CTLA-4 and PD-L1 blockade were combined with *in vivo* depletion of CD4^+^ or CD8^+^ cells (**Figure 6**). Depletion of CD8^+^ cells before and during *Pb*A infection (α-CD8 antibodies administered on days -1, 0, +4 and +6 of infection; full course) or just prior to the expected onset of neurological signs (α-CD8 antibodies administered on Days +4 and +6 of infection; late) completely abrogated the development of ECM in both α-CTLA-4-treated (**Figure 6A** **)** and α-PD-L1-treated (**Figure 6B** **)** mice; instead, CD8-depleted mice developed severe anaemia and were euthanised significantly later than non-depleted mice.

In contrast, although depletion of CD4^+^ cells throughout infection (α-CD4 antibodies administered on days −1, 0, +4 and +6 of infection) led to a delay in onset of ECM in α-CTLA-4 treated mice (day 8–11 as compared to day 7–8 in α-CTLA-4 treated mice but were not given α-CD4 antibodies), CD4^+^ depletion later in infection (α-CD4 antibodies administered on days +4 and +6 of infection) in α-CTLA-4 treated mice had no effect on the development of ECM. These results are entirely consistent with data from C57BL/6 mice indicating that CD4^+^ T cells play an essential helper role in priming CD8^+^ T cells in the first 4 or 5 days of infection but after this time only CD8^+^ T cells are required for initiation of ECM [40].

Among α-PD-L1 treated mice, neither CD4^+^ depletion throughout infection nor CD4^+^ depletion later in infection had any significant effect on the development of ECM. These results reveal fundamental differences between the CTLA-4 and PD-1 pathways and suggest that the CD4^+^ T cells in this infection model are effectively regulated via the CTLA-4/B-7 pathway but are relatively unaffected to regulation via the PD-1/PD-L1 pathway. Moreover, additional pairwise analysis comparing the results of CTLA-4 blockade and PD-L1 blockade in Figure 6 indicate that the effects of CTLA-4 blockade and PD-L1 blockade are similar (i.e. not significantly different) for all treatment regimes with the exception that CTLA-4 blockade combined with late-stage CD4 depletion is significantly different than is PD-L1 blockade combined with late-stage CD4 depletion (p = 0.0006; Bonferroni correction indicates that the threshold for significance is p = 0.006).

Both IFN-γ and TNF have been implicated in the development of ECM [38], [58]. To determine their roles in the induction of ECM in α-CTLA-4- and α-PD-L1-treated *PbA*-infected BALB/c mice, *in vivo* treatment with neutralising α-IFN-γ or α-TNF antibodies throughout infection (days −1, 1, 3, 5, 7) was performed (**Figure 6** **C,D**). Among α-CTLA-4 treated mice, neutralisation of IFN-γ abrogated the development of ECM, while neutralisation of TNF had a small but significant effect on the survival curve. Among the α-PD-L1 treated mice, neutralisation of IFN-γ also abrogated the development of ECM, but neutralisation of TNF had no significant effect.

## Discussion

Regulation of effector T cell function is crucial for immune homeostasis during infection. Immune homeostasis is maintained, in part, by the negative regulators of T cell activation, CTLA-4 and PD-1. While PD-1/PD-L1 - and to some extent the CTLA-4/B-7 pathway - negatively regulate T cell responses during chronic infections, their roles in acute infections are much less clear. In addition, there is limited information available for the role of PD-1/PD-L2 during infection. The extent to which these pathways may regulate different aspects of the T cell response to acute infections in animals with differing susceptibility to immunopathology is not known. Whilst other pathways of immune regulation may contribute to the outcome of virulent malaria infections, only modest effects on *Pb*A-induced ECM were observed after IL-10 neutralisation in BALB/c mice [59]. Similarly, depletion of regulatory T cells using α-CD25 antibodies did not increase mortality rates in BALB/c mice during primary infection with *Pb*A [60]. Therefore, in this study, we directly compared the roles of the CTLA-4 and PD-1/PD-L pathways during acute infection with a virulent rodent malaria parasite, *PbA* in ECM-susceptible (C57BL/6) and ECM-resistant (BALB/c) mice.

We initially hypothesised that inadequate T cell expression of CTLA-4 and PD-1 during infection, leading to overproduction of Th-1 cytokines and migration of activated CD8^+^ T cells to the brain, could explain susceptibility to ECM. Interestingly, contrary to our expectations, proportions of CD4^+^ and CD8^+^ T cells expressing CTLA-4 and PD-1 were significantly higher in ECM-susceptible C57BL/6 mice than in ECM-resistant BALB/c mice, and CTLA-4 and PD-1 expression were positively correlated with IFN-γ secretion as well as with the CD11a^hi^ and CD62L^lo^ phenotype. Indeed, CTLA-4 and PD-1 expression coincided with expression of the activation markers CD11a^hi^ and CD62L^lo^. The expression of both CD11a^hi^ and CD62L^lo^ were used as surrogate T cell activation markers due to a paucity of defined CD8^+^ or CD4^+^ T cell epitopes associated with pathological responses. Besides the expansion of parasite-specific T cells, there is likely to be non-specific bystander activation. However, it remains unclear whether T cells activated in a bystander manner can contribute to ECM pathogenesis. Studies with ovalbumin-expressing *Pb*A and ovalbumin-specific transgenic T cells suggest that this is possible since transfer of antigen-specific effector memory cells into mice deficient of recombination-activating gene is not always sufficient to induce ECM [61], [62]. Indeed, in their study, Miyakoda *et al* specifically analysed non-specific activation of CD8 T cells [62] and found that while non-specific activation occurred it was at a much lower level compared to specific activation.

Our results raise the intriguing and important question of why the very efficient activation of CTLA-4 and PD-1 pathways in C57BL/6 mice fails to protect them from acute immune pathology. One interpretation is that T cell activation is so extensive in ECM-susceptible C75BL/6 mice that positive T cell-derived signals override physiological levels of immune inhibition mediated by CTLA-4 and PD-1. Another interpretation of this data is that in ECM-susceptible mice, CTLA-4 and PD-1 are induced on highly activated T cells, but that their down-stream signalling is impaired. Further studies are required to understand why these potent immunoregulatory pathways are unable to control T cell activation in animals with severe malarial immunopathology and to determine whether these pathways play roles in humans with cerebral malaria.

Strikingly, however, *in vivo* blockade of either CTLA-4 or PD-1/PD-L1, but not PD-1/PD-L2, rendered otherwise resistant BALB/c mice fully susceptible to ECM. Treated animals exhibited characteristic neurological signs, their brains revealed the cardinal features of ECM (haemorrhages, CD8^+^ T cell arrest and parasite intravascular accumulation in the microvasculature) and there was clear evidence of excessive systemic inflammation and T cell activation. Thus, CTLA-4 and PD-1/PD-L1 play essential, independent and non-redundant roles in preventing ECM in resistant animals; this reinforces the urgent need to compare the activation and down-stream effects of these pathways in humans with or without severe malaria pathology. It is noteworthy that *in vivo* blockade of either CTLA-4 did not affect the expression of PD-1 on T cells; *in vivo* blockade of PD-1/PD-L1 did not affect the expression of CTLA-4 on T cells (data not shown).

This study reveals – for the first time - evidence of subtle but important differences in the effects of CTLA-4-mediated and PD-1/PD-L1-mediated immune regulation during acute infections. Infected animals treated with α-CTLA-4 always succumb to infection significantly earlier than animals treated with α-PD-L1. In addition, disruption of the CTLA-4 pathway led to higher levels of T cell activation, significantly higher levels of circulating TNF and IL-6 and, accordingly, earlier onset of ECM than did blockade of the PD-1/PD-L1 pathway. Furthermore, we observed that depletion of CD4^+^ T cells during *Pb*A infection abrogated the effects of α-CTLA-4 treatment but had no effect on α-PD-L1 treatment. Thus, our study suggests that CTLA-4 may be much more effective than PD-1/PD-L1 at regulating CD4^+^ T cells particularly in this experimental model of cerebral malaria, although this hypothesis remains to be directly tested. Since CD4^+^ T cells are essential for activation of CD8^+^ T cells and for their arrest in the brain during ECM [40], effective regulation of CD4^+^ T cells by CTLA-4 is likely to interrupt the chain of events leading to ECM at a much earlier stage of infection than is regulation of CD8^+^ T cell activity (by either CTLA-4 or PD-1/PD-L1). It should be noted however, that in other models - such as during *M. tuberculosis* infection - the PD-1/PD-L1 pathway has a direct effect on CD4^+^ T cells in preventing T cell-driven exacerbation of infection [63].

Our study thus raises the intriguing hypothesis that in the our malaria model, CTLA-4 may be the primary regulatory pathway for CD4^+^ T cells, thereby indirectly affecting CD8^+^ T cell responses, whereas PD-1/PD-L1 preferentially and directly fulfils this role for CD8^+^ T cells. This notion is consistent with published data suggesting that CD8^+^ T cells are less dependent upon costimulation through the CD28/CTLA-4/B7 axis than are CD4^+^ T cells [64]-[66] and that blockade of the PD-1/PD-L1 pathway restores the effector functions of CD8^+^ T cells in the absence of CD4^+^ T cell help [16], [67]. Such a hypothesis is also consistent with associations between PD-1/PD-L1 expression and inability to control chronic viral infections such as LCMV [16] and rabies [30], the failure of CTLA-4 blockade to ameliorate CD8^+^ T cell exhaustion during LCMV infection [16], and the preferential expression of CTLA-4 on CD4^+^ rather than CD8^+^ cells during HIV infection [17]. In addition, in LCMV-infected mice that lacked CD4^+^ T cell-help, blockade of the PD-1/PD-L1 pathway reinvigorated the ‘helpless’ CD8^+^ T cells and allowed them to function as effector cells [16].

Our observation that CTLA-4 blockade enhances susceptibility to ECM in BALB/c mice but had no effect on the outcome of infection in C57BL/6 mice is somewhat at odds with a previous study in which CTLA-4 blockade was shown to increase susceptibility to ECM, but in C57BL/6 mice [68]. The apparent discrepancy may be explained by the unusually slow kinetics of *Pb*A infection in the study of Jacobs *et al* and by the fact that the majority of the control mice survived for at least 20 days after infection and did not develop ECM [68]; in our hands, and those of most other investigators, *Pb*A uniformly causes death from ECM within 10 days in all C57BL/6 mice. It is not possible to distinguish whether this reflects differences in susceptibility between colonies of C57BL/6 mice or differences in virulence of the parasite isolates (the *Pb*A parasites used in the study of Jacobs *et al* appear to be highly attenuated), but the message is clear: the virulence of mouse/parasite combinations that do not normally lead to ECM is significantly exacerbated by CTLA-4 blockade.

In addition to the very important observations emanating from our direct side-by-side comparison of the roles of CTLA-4 and PD-1/PD-L1, we have significantly extended our understanding of the role of T cell regulatory pathways during malaria infection by thoroughly characterising the effects of regulatory blockade on both CD4^+^ and CD8^+^ T cells. The onset of ECM in BALB/c mice following CTLA-4 or PD-1/PD-L1 blockade was accompanied by elevated production of pro-inflammatory cytokines and by increased migration of activated CD8^+^ T cells to the brain. Neutralisation of IFN-γ or depletion of CD8^+^ T cells during *Pb*A infection was shown to reverse the pathologic effects of the inhibitory pathway blockade, confirming that the aetiology of ECM in the BALB/c mice is similar to that in susceptible C57BL/6 mice [40]. These experiments not only identify the primary targets of CTLA-4- and PD-1/PD-L1-mediated regulation as being pro-inflammatory T cells, but also re-emphasise that CD8^+^ T cells and IFN-γ are critical effectors of ECM.

In summary, we have revealed essential, independent and non-redundant roles for CTLA-4/B-7 and PD-1/PD-L1 pathways in regulating T cell-mediated pathology and host resistance to *Pb*A-induced ECM. Exploration of the relationship between T cell regulatory pathways and outcome of malaria infection in humans is clearly now a priority; such studies will need to go beyond simple characterisation of CTLA-4 or PD-1 expression [69], [70] and consider the potential for genetic variation in the receptors, their ligands and downstream signalling molecules to affect the outcome of infection.

## Supporting Information

Text S1 **Compilation of supplemental Protocol and Figures (S1-S5).**
(DOC)
