# Supplementary material for: The CTLA-4 and PD-1/PD-L1 Inhibitory Pathways Independently Regulate Host Resistance to Plasmodium-induced Acute Immune Pathology
Source: PLoS Pathog. 2012 Feb 2;8(2):e1002504. doi: 10.1371/journal.ppat.1002504 (PMC3271068; doi:10.1371/journal.ppat.1002504)
Supplement: Text S1 — Compilation of supplemental Protocol and Figures (S1-S5). (DOC) [file ppat.1002504.s001.doc]

**Supplementary Information for:**

**The CTLA-4 and PD-1/PD-L1 inhibitory pathways independently regulate host resistance to *Plasmodium*-induced acute immune pathology**

Julius Clemence R. Hafalla^a,1^, Carla Claser^b^, Kevin N. Couper^a^,

Georges Emile Grau^c^, Laurent Renia^b^, J. Brian de Souza^a,d,2^

and Eleanor M. Riley^a,2^

^a^ Department of Immunology and Infection, Faculty of Infectious and Tropical Diseases, London School of Hygiene and Tropical Medicine, Keppel Street, London WC1E 7HT, United Kingdom

^b^ Laboratory of Malaria Immunobiology, Singapore Immunology Network (SIgN), Agency for Science, Technology and Research (A*STAR), 8A Biomedical, Immunos, Grove, Biopolis, Singapore 138648

^c^ Vascular Immunology Unit, Department of Pathology, School of Medical Sciences, Bosch Institute, The University of Sydney, New South Wales 2006, Australia

^d^ Division of Infection and Immunity, University College London Medical School, The Cruciform Building, Gower Street, London WC1E 6BT, United Kingdom

^1^ Corresponding Author:

Dr. Julius Hafalla, Department of Immunology and Infection, Faculty of Infectious and Tropical Diseases, London School of Hygiene and Tropical Medicine, Keppel Street, London WC1E 7HT, United Kingdom; Phone +44 20 7927 2462, Fax +44 20 7637 4314,

E-mail [Julius.Hafalla@lshtm.ac.uk](mailto:Julius.Hafalla@lshtm.ac.uk)

^2^ These authors contributed equally to this work.

**Contents:**

- Supplemental Protocol
- Supplemental Figures 1 to 5 (S1 to S5)

**Hafalla *et al*., Protocol S1**

**Supplementary Materials and Methods**

Animal experimentation legislation in the United Kingdom prohibits unnecessary suffering, we are required to kill the animals as soon as they show irreversible signs compatible with a diagnosis of CM. To do this we use the following clinical staging scheme: (1) no signs, (2) ruffled fur and/or abnormal posture and/or weight loss < 15% of body weight, (3) lethargy and/or weight loss < 20%, (4) reduced response to stimulation and/or respiratory distress/hyperventilation, and/or weight loss > 20% (5) prostration and/or ataxia and/or paralysis, and/or weight loss > 25%, and (6) death. Stage 5 is diagnostic of ECM; mice are killed as soon as they reach this stage and in all cases the clinical diagnosis is confirmed post mortem. Survival curves and cumulative ECM incidence are based on the proportion of animals showing ataxia and/or paralysis in stage 5.

**Hafalla *et al*., Figure S1**


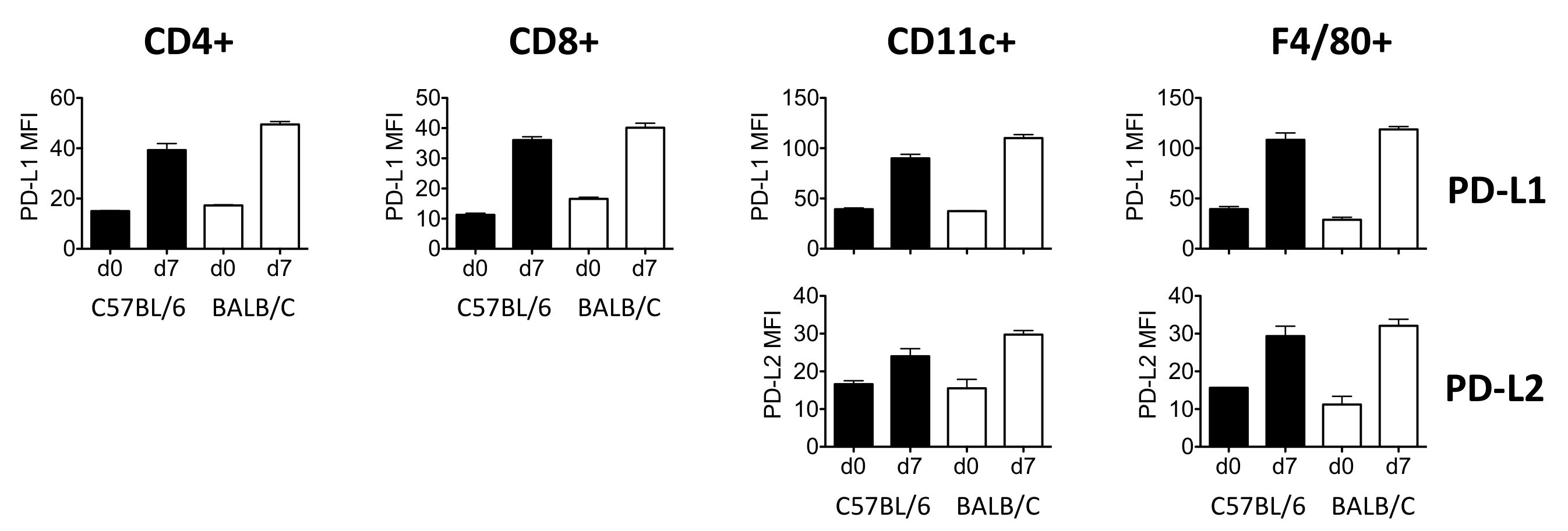


**Figure S1. Upregulation of PD-L1 and PD-L2 during *PbA* infection of C57BL/6 and BALB/C mice**

Mice were infected i.v. with 10^4^ *PbA*. Splenocytes were prepared from uninfected (d0) or day 7 infected mice and stained for surface CD4, CD8, CD3, CD11c and F4/80. Mean fluorescence intensities (MFI; mean + SD) are shown. Results are representative of at least three similar experiments (3-5 mice per group per each experiment).

**Hafalla *et al*., Figure S2**


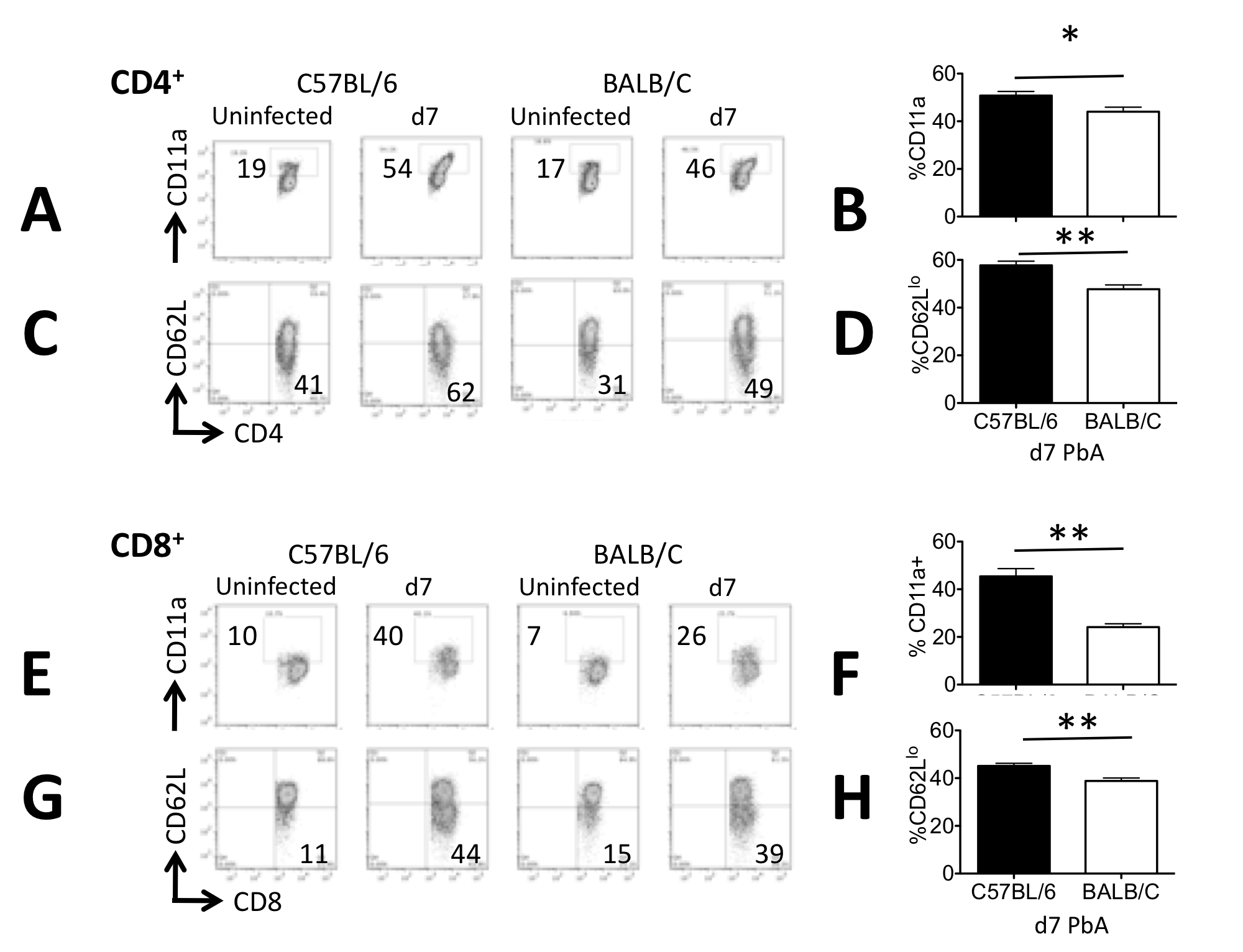


**Figure S2. *Pb*A-infected C57BL/6 mice have higher proportions of T cells with activated phenotype (CD11a^hi^ and CD62L^lo^)**

than BALB/C mice. Mice were infected i.v. with 10^4^ *PbA*. Splenocytes were prepared from uninfected (d0) or day 7 infected mice and stained for surface CD4 (A-D) or CD8 (E-H), CD11a and CD62L. Representative FACS plots showing (A, E) CD11a^hi^ and (C,G) CD62L^lo^ populations of T cells. (B,F) Comparison of the proportions of CD11a^hi^ T cells. (D,H) Comparison of the proportions of CD62L^lo^ T cells. Results are from a representative of at least three similar experiments (3-5 mice per group per experiment). P-values are based on Mann Whitney test.

**Hafalla *et al*., Figure S3**


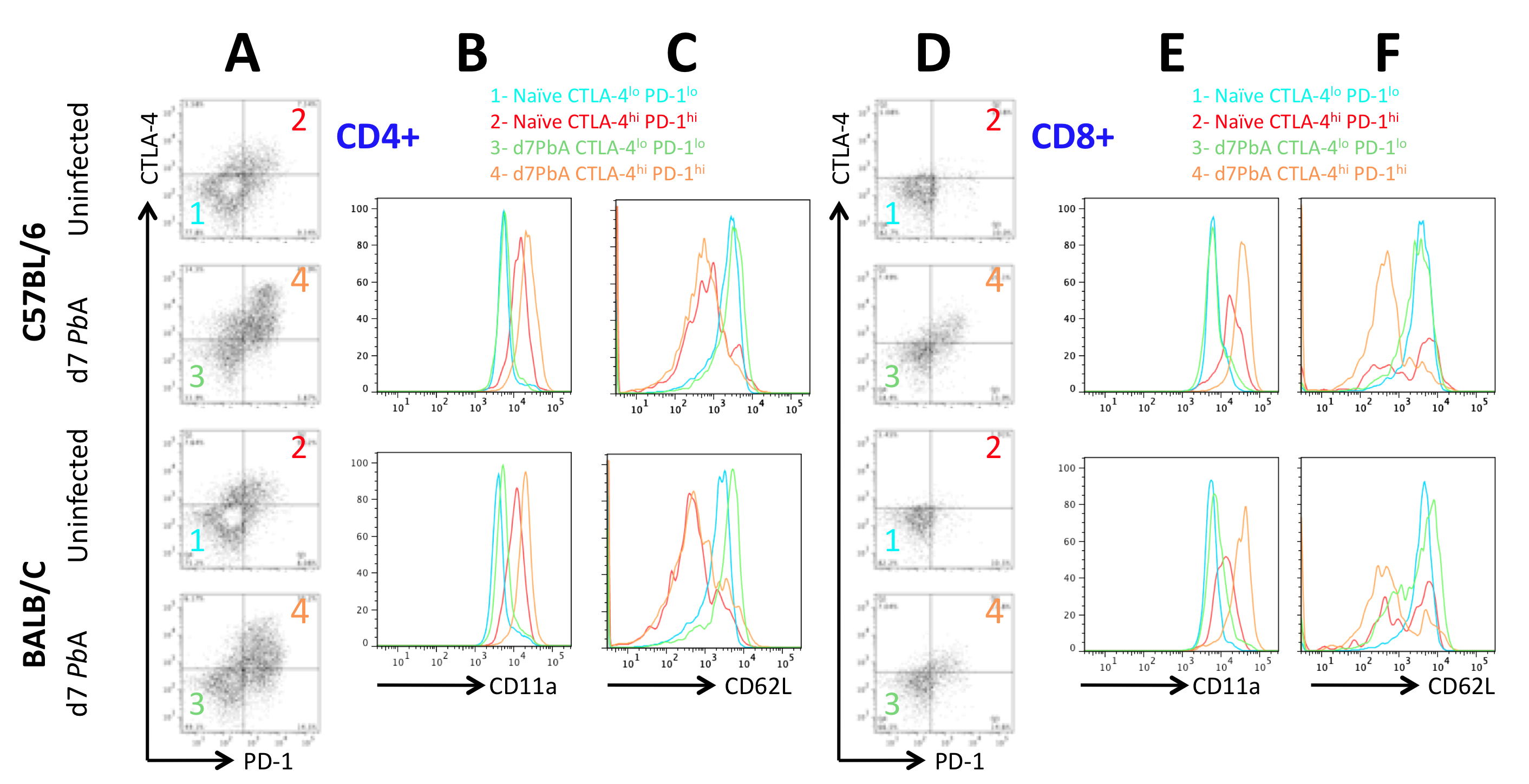


**Figure S3. CTLA-4^hi^/PD-1^hi^ expression coincide with markers of effector cells: CD11a^hi^ CD62L^lo^**

Mice were infected i.v. with 10^4^ *PbA*. Splenocytes were prepared from uninfected or day 7 infected mice and stained for CD4 or CD8 and surface CD11a, CD62L and PD-1. CTLA-4 staining for both surface and intracellular protein was included. (A,D) Plots of CTLA-4 versus PD-1 after gating on lymphocytes and (A) CD4 and (D) CD8. (B,E) Histogram plots for CD11a. (C,F) Histogram plots for CD62L.

**Hafalla *et al*., Figure S4**

**
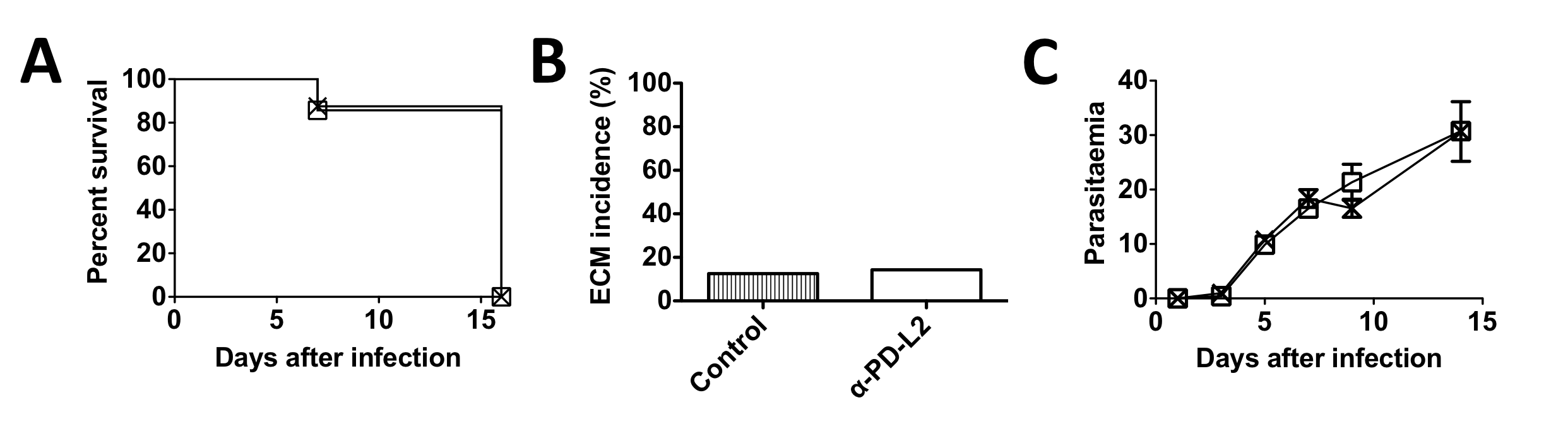
**

**Figure S4. PD-L2 blockade in *Pb*A-infected BALB/c mice does not alter the course of infection and disease.**

BALB/c mice were infected i.v. with 10^4^ *PbA* and treated with α-PD-L2 antibodies or with no antibody (control). **(A)** Survival curve. X = Control (n=8); 🞎 = α-PD-L1 (n=7). **(B)** Percentage of mice developing experimental cerebral malaria. **(C)** Parasitaemia levels, shown as mean + SD, of *PbA*-infected mice.

**Hafalla *et al*., Figure S5**

**
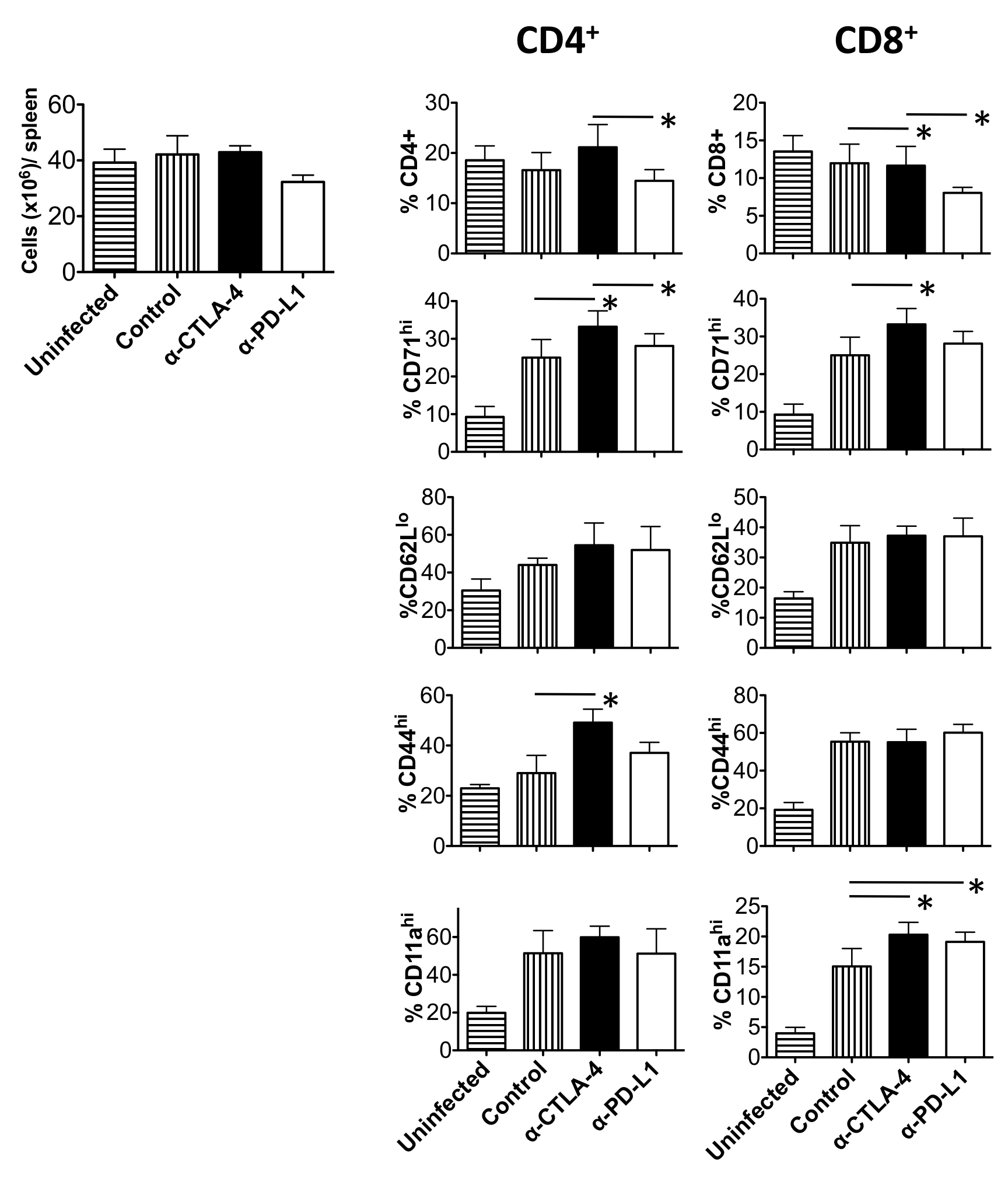
**

**Figure S5. Enhanced effector responses following CTLA-4 and PD-L1 blockade.** Mice were infected i.v. with 10^4^ *PbA*. Splenocytes were prepared from uninfected (d 0) or day 7 infected mice and stained for surface CD4, CD8, CD71, CD62L, CD44 and CD11a. Proportions (mean + SD) of surface marker positive cells (negative for CD62L) are shown. Results are representative of at least two similar experiments (3-5 mice per group per each experiment), * P < 0.05, Kruskal-Wallis Test/Dunn’s multiple comparison test. Note that control mice refer to those treated with rat IgG or PBS or left untreated.
